# Measuring Daylight: A Review of Dosimetry in Daylight Photodynamic Therapy

**DOI:** 10.3390/ph12040143

**Published:** 2019-09-20

**Authors:** Paul O’Mahoney, Marina Khazova, Ewan Eadie, Sally Ibbotson

**Affiliations:** 1 Photobiology Unit, NHS Tayside, Ninewells Hospital, Dundee DD1 9SY, UKshibbotson@dundee.ac.uk (S.I.); 2 The Scottish Photodynamic Therapy Centre, Dundee DD1 9SY, UK; 3 School of Medicine, University of Dundee, Dundee DD1 9SY, UK; 4 Public Health England, Didcot OX11 0RQ, UK; marina.khazova@phe.gov.uk

**Keywords:** daylight, photodynamic therapy, dosimetry, actinic keratosis, sunscreen

## Abstract

Successful daylight photodynamic therapy (DPDT) relies on the interaction of light, photosensitisers and oxygen. Therefore, the ‘dose’ of light that a patient receives during treatment is a clinically relevant quantity, with a minimum dose for effective treatment recommended in the literature. However, there are many different light measurement methods used in the published literature, which may lead to confusion surrounding reliable and traceable dose measurement in DPDT, and what the most appropriate method of light measurement in DPDT might be. Furthermore, for the majority of practitioners who do not carry out any formal dosimetry and for the patients receiving DPDT, building confidence in the evidence supporting this important treatment option is of key importance. This review seeks to clarify the methodology of DPDT and discusses the literature relating to DPDT dosimetry.

## 1. Introduction

**Figure 1:**
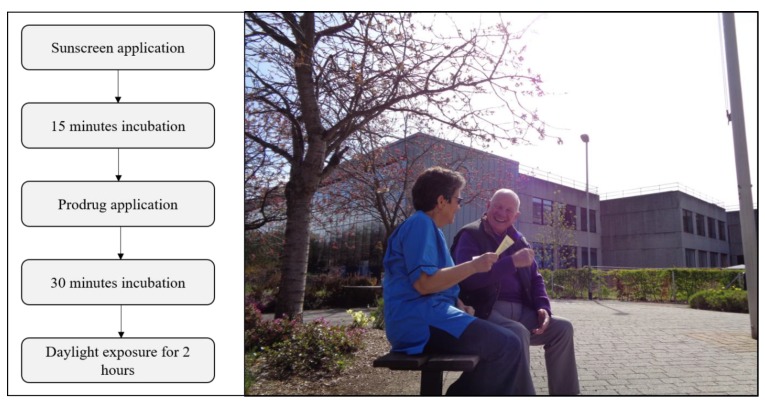
(**Left**): flowchart of the successful daylight photodynamic therapy (DPDT) protocol. (**Right**): patient receiving information before DPDT at Ninewells Hospital, Dundee.

Daylight photodynamic therapy (DPDT) is an effective, convenient, almost painless and patient-preferred option for the management of field-change actinic keratoses (AK) [1,2,3,4,5,6]. The exact nature of field-change may not be precisely defined [7],and it is often at the discretion of the clinician, based on a clinical examination, whether small areas or entire fields are to be treated. Treatment involves application of sunscreen to limit ultraviolet-induced erythema and application of a photosensitiser pro-drug to the affected areas prior to exposure to continuous daylight for at least two hours (Figure 1). The essential step in daylight photodynamic therapy is light activation of tissue-localised photosensitiser (protoporphyrin-IX; PpIX) in the presence of molecular oxygen. As drug, light and oxygen are all necessary, it stands to reason that deficiency in any one of these three components may reduce the efficacy of treatment. Early studies suggested that a minimum threshold “effective light dose” was required during DPDT in order to achieve effective treatment outcomes: above this threshold dose, there was no additional dose-dependent therapeutic benefit gained and below this threshold, there was a significant reduction in therapeutic efficacy [8,9]. Thus, as the light exposure received by patients during DPDT is such a key component to deliver effective treatment, understanding the nature of this exposure, its measurement and influencing factors are of critical importance with respect to providing useful information for the DPDT practitioner and patient while improving reassurance and confidence in the use of this important therapeutic option. Most studies investigating DPDT do not measure light exposure and those that do report a range of measurement techniques and often do not measure at the treatment site, leading to an inconsistency of dosimetry in DPDT. In this review, we highlight the dosimetry methods that have been applied in DPDT studies and outline the key differences, including advantages and disadvantages, between these methods and outline the current best practice based on existing literature and expertise.

## 2. Methodology and Literature Searches

The standard licenced protocol for DPDT comprises several steps [10,11]. A high SPF (>30) sunscreen with no inorganic filters (titanium dioxide, zinc oxide, iron oxide) should be applied to the entire sun exposed area and be allowed to dry for 15 min. Scales and crusts should be removed and the lesion surface gently roughened, most commonly with curettage. A photosensitiser pro-drug formulation is then applied to the lesions. Currently, Metvix^®^ (Galderma, Hertfordshire, UK) and Ameluz^®^ (Biofrontera, Leverkusen, Germany) are licenced for use in DPDT of AKs in Europe. Within 30 min after application of pro-drug, two hours of daylight exposure should commence, with the cream removed after daylight exposure has finished. There is scant guidance on light exposure dose in the licensed protocol, stating only that treatment should be avoided if weather conditions are or are likely to become rainy. In the European guidelines, there are more details concerning the evidence surrounding light exposure [2]; however, it is important to note that there is no requirement to carry out dosimetry during DPDT. As such, many DPDT studies, clinics and practitioners do not carry out any light measurements.

A literature search of PubMed and Embase for “daylight photodynamic therapy” revealed 171 results (1 January 2008–18 July 2019, all study types included); however, manual screening of these publications showed there were only twelve clinical studies of DPDT in which dosimetry was undertaken, whilst an additional five studies carried out a location-based analysis of DPDT dosimetry (Table 1).

### 2.1. Quantifying Dosimetry in Daylight PDT

**Figure 2:**
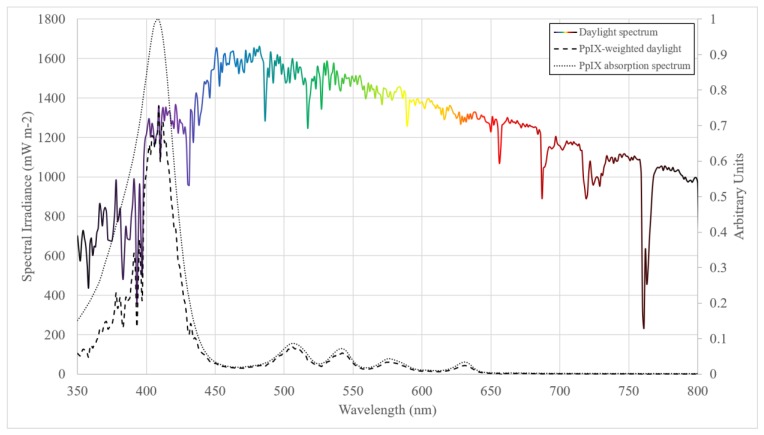
Multicoloured line: example daylight spectrum (12:20 h, 8 June 2019, Chilton UK). The relative spectral content of the daylight spectrum changes with the position of the sun in the sky and atmospheric conditions. Short dash line: the absorption spectrum of PpIX [25]. Long dash line, the PpIX-weighted spectral irradiance of daylight.

The term “dose” has no official definition when it comes to DPDT. The “dose” referred to in many DPDT studies is actually the PpIX-weighted radiant exposure (measured in joules per centimetre squared; J cm^−2^), which is the PpIX-weighted irradiance integrated over the treatment duration [8]. PpIX-weighted irradiance is the product of the spectral irradiance of daylight and the PpIX absorption spectrum (normalised between 0 and 1), integrated over the required wavelength range (Figure 2). There are various terms used throughout the DPDT literature pertaining to dosimetry, and we outline the definitions here to clarify:

- Irradiance (W m^−2^) is the radiant flux received by a surface per unit area, where radiant flux (W, commonly called ‘power’) is the electromagnetic energy received per unit time.
- Spectral irradiance (W m^−2^ nm^−1^) is the irradiance per unit wavelength and gives spectral information about a light source, which is important when considering the application of broadband light sources in the medical setting and weighting for functions such as PpIX absorption.
- Radiant exposure (J m^−2^) is the irradiance received by a surface integrated over a set duration. In DPDT radiant exposure is given in the units J cm^−2^ and, when the irradiance is weighted for PpIX absorption, may be referred to as either ‘PpIX-weighted dose’, ‘PpIX-effective dose’ or simply ‘PpIX dose’.
- Illuminance (lumens m^−2^; lx) is the photometric equivalent to irradiance after weighting to the luminosity function—the perception of the human eye to the light brightness. Defined as the luminous flux (lumens) received by a surface per unit area.
- Intensity and brightness should not be used for quantitative measurements so should be avoided where possible in the context of DPDT dosimetry.

There is understandable confusion in the literature surrounding the application of these terms in DPDT dosimetry, and it is one of the aims of this review to clarify the use of these definitions in DPDT clinical practice. The goal in DPDT dosimetry is to quantify the dose received by the diseased tissue; however, what we often determine and refer to is dose incident at the measurement site. This also pertains to the orientation of the measurement site and lesion relative to direct daylight, as differences here may cause further inconsistencies in measurements of dose. Though measurement of the ‘true’ dose is feasible, it is rarely practical given available measurement methods.

Different PpIX absorption spectra exist, and these vary depending on the method by which they are measured. As shown by Marra et al., using different PpIX absorption spectra in calculations on the same spectral irradiance can lead to different PpIX-effective doses [26]. Therefore, it is important to maintain traceability of both the light source and PpIX absorption spectra used in dose assessment. It is worth noting here that the PpIX absorption spectrum is not necessarily the action spectrum for DPDT efficacy. Such an action spectrum has not been reported in the literature, and as such, the absorption spectrum of PpIX was used as a surrogate which allows relative comparability between light sources. Although care should be taken when comparing PpIX-effective light doses from light sources with vastly different spectra, as such, comparisons become less meaningful clinically due to wavelength-dependent processes, such as skin penetration, and other wavelength-dependent photoproducts and absorption peaks which may also be relevant due to the broadband coverage of the daylight spectrum.

Furthermore, in the literature, clinical dosimetry in DPDT almost exclusively refers to dose-to-skin surface. Thus, for all clinical studies reporting dose, no direct inferences can be made in terms of dose at specific skin depth or on attenuation due to skin pigmentation. Indeed, PpIX activation within the skin may vary between patients and between individual lesions, which dose-to-the-skin surface dosimetry cannot discriminate between. However, depth-dependent dosimetry in DPDT has been modelled with MCRT and it was shown that DPDT may remain effective at depths of 2 mm for a 2.5 h exposure time on a cloudy day [27]. Therefore, with the aid of computer modelling, doses may be estimated below the skin surface, including the impact that wavelength-dependent attenuation of daylight at such depths may have on PpIX-effective light dose. Vignion-Dewale et al. used mathematical modelling to demonstrate local damage in tissue for ten different PDT modalities and found that DPDT may have as much local damage (singlet oxygen generation) and PpIX-effective light dose delivered as conventional PDT regimens at a depth of 100 µm [28]. Actinic keratoses are superficial and are thus equally treatable with either less penetrating blue or more deeply penetrating red light during PDT [29]. In addition, given the complexity surrounding in vivo dose-to-diseased target tissue as opposed to dose-to-normal skin surface dosimetry, surface dosimetry measurements are generally accepted as suitable proxy for DPDT.

### 2.2. Dosimetry Methods Reported in the Literature

#### 2.2.1. Measurements of Spectral Irradiance

Irradiance is a radiometric measurement and thus has no weighting for any efficacy or absorption spectra. By measuring the spectral irradiance of daylight, it is possible to then weight for the PpIX absorption spectrum and to integrate over the known treatment duration to obtain PpIX-effective light dose. There are generally two types of devices used to measure spectral irradiance: a scanning spectroradiometer and an array spectrometer. The double grating scanning spectroradiometer is considered the gold standard for spectral irradiance measurements of light sources and introduces the least measurement error. However, scanning systems suffer a slow acquisition rate and are generally less portable than array spectrometer systems. Array spectrometers are typically much more compact, often cheaper and are capable of acquisition times << 1 s. Although stray light and temperature dependence of performance may reduce accuracy and reliability. Any measurement device should have traceable calibration to national standards (NIST USA, NPL UK, PTB Germany, LNE France). Measurements should be taken throughout treatment to capture the changing levels of daylight and could then be time-integrated over the treatment duration in order to obtain a more representative PpIX-effective light dose.

In a multi-centre Australian study [15], spectral irradiance was measured with an array spectrometer (ILT950, International Light Technologies, Peabody, MA, USA) and the PpIX-weighted dose was then calculated. The measurement intervals during the patient’s treatment and the PpIX absorption spectrum used were not specified and thus, there was lack of clarity in the methodology. However, patients (*n* = 90) were reported to have received PpIX-effective light doses of 22.8 ± 12.4 J cm^−2^ (range 3–46), using the array spectrometer measurements as a surrogate for patient received dose, with no correlation found between PpIX-effective light dose and treatment efficacy. It is reported in the European guidelines [2] that in a follow-up European study [4], mean irradiance during a two-hour DPDT exposure was 267 W m^−2^ (range 44–601), measured by spectroradiometer. In that study, measurements were not weighted for PpIX, nor were they used to estimate a patient received dose, although an inference was made by the authors that 130 W m^−2^ was equal to a PpIX-effective light dose of 8 J cm^−2^, although this was an approximation. Crucially, the wavelength range of the spectral irradiance measurements defined was not reported in either of those studies, which makes any unweighted irradiance values difficult to compare reliably to a PpIX-effective light dose.

In a separate study comparing the efficacy of DPDT and artificial white light PDT, O’Gorman et al. measured spectral irradiance of daylight [18]. Specifics of the devices were reported in a subsequent publication from the same group [30], and the PpIX absorption spectrum used in dose calculations was not specified. The authors used spectral measurement as a surrogate for patient-received radiant exposure and reported that patients (*n* = 20) received a mean of 21.38 ± 13.25 J cm^−2^ (range 3.2–43), with no correlation between light dose and treatment efficacy. It is also important to note that in that study, the devices were placed in the horizontal plane next to patients during treatment and therefore, these dose measurements are likely to be more representative than if patients had left the hospital grounds during treatment.

#### 2.2.2. Broadband Measurements

Due to cost and restricted portability, spectral measurement is often impractical. It is also impersonal and not available at the patient’s home-based location. Spectrometers often need to be attached to a computer in order to take measurements, receive power and interpret data. Additionally, spectrometers are high-cost equipment and thus, relatively impractical when compared to cheaper alternatives such as radiometers or luxmeters. This is perhaps exemplified by the relative lack of reported clinical studies which employed measured spectral irradiance.

Broadband radiometers are often handheld, relatively cheap instruments which measure electrical signal from a photodiode which, when appropriately calibrated, can be converted to irradiance. They provide no spectral information, but their response is spectrally dependent and therefore, they must be calibrated against the light source being measured in order to provide accurate and reliable irradiance readings. Calibrating a radiometer against daylight, however, is problematic as the spectrum of daylight changes significantly depending on the position of the sun in the sky, cloud cover and atmospheric conditions, e.g., humidity, and therefore, there will always be inherent uncertainty using this method.

Grinblat et al. used a fixed position radiometer in their 2014 study [16]. The radiometer measured values every 5 min and the measurement of radiant exposure was taken as a surrogate for mean PpIX-weighted dose measured, which was 28.3 J cm^−2^ (range 7.9–45.3), with no relationship observed between dose and efficacy. It is not clear how the relationship between measured irradiance (PBW, Eppley Laboratory, Newport, RI, USA) and PpIX-effective light dose was made in this study, as irradiance and PpIX irradiance (hence PpIX-effective light dose) are not readily interchangeable.

Räsänen et al. used a broadband radiometer fitted with a blue light hazard sensor (UV-3709-4, Gigahertz-Optik, Germany) [20]. The authors stated that the design of the blue light hazard sensor allows ‘mimicking’ of the PpIX absorption spectrum in order to directly measure PpIX irradiance, perhaps overcoming some of the limitations with regards to the interchangeability of irradiance and PpIX irradiance. The instrument is calibrated with a spectroradiometer as the reference instrument in order to measure PpIX-weighted light irradiance, however, the responsivity of the sensor used is not identical to the PpIX absorption function. It seems that the objective is to capture the Soret band of the PpIX absorption spectrum (~408 nm) and some of the lower wavelength Q-bands (<550 nm), however, the higher wavelength Q-bands (>550 nm) are not captured by a detector with this responsivity and wavebands in between the absorption peaks of PpIX are over represented. Additionally, the responsivity at the lower wavelength range begins to drop off drastically at ~420 nm, which may then result in underrepresentation of the Soret band of PpIX. Crucially, the spectrum of daylight, particularly in the Soret band, changes with varying conditions, such as cloud cover, aerosols content, and the position of the sun in the sky, giving rise to greater challenges in the calibration of such a device. Ultimately, these discrepancies may lead to an inaccurate equivalence between the measured value and the true PpIX-weighted irradiance. The idea of a detector that ‘mimics’ PpIX absorption may lead to overconfidence in measurements unless calibration to daylight appropriate for the time of the day, season and weather conditions is performed, or modification of spectral responsivity for very precise mimicking of the PpIX absorption spectrum is made. Nevertheless, the authors measured mean PpIX-effective light dose of 12.89 J cm^−2^ (range 5.96–17.93 J cm^−2^) with no relationship between dose and clearance rate.

Illuminance is the visually perceived brightness of light as defined by the luminosity function on a surface per unit area. It is the photometric equivalent of the radiometric quantity, irradiance. The luminosity function describes the perception of light by the human eye and peaks at 555 nm [31]. While in general, this is a useful quantity to measure in lighting applications where perceived brightness (luminous flux; lumens) is a key requirement, it is perhaps less useful in the context of photodynamic therapy where the PpIX absorption spectrum is key. However, measurement of illuminance is very accessible—cheap handheld luxmeters are ubiquitous. This makes measurement of illuminance a very attractive method for measuring visible light levels and potentially PpIX-effective light dose if appropriate calibrations and conversions are made.

In the first study on DPDT by Wiegell et al. [32], illuminance on a handheld luxmeter measured periodically during treatment was used to correct simulated spectral irradiance into PpIX-effective light dose. Once more, it is emphasised that the measured dose is not the same as the patient received dose, which underlies the importance of clear, defined methodology in dose measurements in DPDT. The spectral irradiance was determined from satellite measurements of cloud cover and atmospheric conditions combined with the luxmeter reading in a radiative transfer model (Danish Meteorological Institute). The resultant spectral irradiance may then be weighted for PpIX and integrated over the treatment duration to receive PpIX-effective light dose. The mean PpIX-weighted dose measured in this study was 43.2 J cm^−2^ (range 11.7–65.9). The patients (*n* = 30) received treatment in the hospital garden and only on days with clear skies.

The subsequent second study used the same methodology but with illuminance readings obtained from a wristwatch luxmeter [8]. This wristwatch was able to collect readings every 5 min, allowing for a more temporally accurate dose calculation. In this study, the mean PpIX-effective light dose was 30.1 J cm^−2^ (range 1.2–69.8). This study is one of only two to demonstrate a minimum threshold PpIX-effective light dose of light for effective treatment (8 J cm^−2^). In measuring illuminance, it is desirable to have the spectral sensitivity of the detector closely resemble the luminosity function, as this will produce the most accurate readings. Many cheaper luxmeters may not have such a response, and have the traditional silicon photodiode response [33] and this ought to be factored into calibrations.

Togsverd-Bo et al. measured illuminance using a luxmeter during a trial investigating the application of ablative fractional lasers in DPDT [17]. In this study, a median illuminance of 48,773 lx (range 10,600–86,357) was measured. This was linked to previous studies [1,8,9] noting that 10,000 lx corresponded to a PpIX-effective light dose of 8 J cm^−2^. Therefore, all patients received above the previously published “minimum dose”, over at least 2 h of daylight exposure. Here, illuminance itself was deemed sufficient as a metric for DPDT dosimetry.

A study by Heerfordt and Wulf in 2019 investigating curettage procedures before DPDT used a similar rationale for dosimetry [21]. Illuminance was measured on a luxmeter (Model E2; Hagner, Solna, Sweden) prior to and at the end of the two-hour treatment. Similarly, Nissen et al. recorded illuminance (Luxmeter 540, Testo AG, Lenzkirch, Germany) prior to and at the end of two-hour daylight exposure in a study comparing the effectiveness of pre-treatment with 5-fluorouracil (5-FU) [19]. This study reported illuminances as low as 1322 lx (mean 36,477 lx, range 1322–94,234 lx) yet clearance was still independent of illuminance in both studies. Significant variations in daylight over a two-hour period are likely, therefore, such a methodology may not give an accurate representation of the daylight exposure received by a patient. As such, it is difficult to draw firm conclusions on the interplay of PpIX-effective light dose and efficacy based on studies using this methodology.

Wenande et al. monitored illuminance on a luxmeter (Luxmeter E2; B Hagner AB, Solna, Sweden) during a trial considering the use of ablative fractional lasers prior to DPDT to ensure that daylight remained above 10,000 lx [34]. This may be a convenient and practical method of ensuring that measured PpIX-effective light dose during a patient treatment would likely be above the 8 J cm^−2^ minimum dose threshold, however, it does not allow for reporting on patient dose.

In 2017, Cordey et al. published their experience of DPDT in Scotland [35]. Previously, a luxmeter was used to measure illuminance prior to treatment, which may not give a good representation of patient received PpIX-effective light dose over the duration of treatment. The authors then implemented personal data-logging light meters worn by the patient as a means of improving the accuracy of patient-specific dosimetry. Important details surrounding the conversion of measured illuminance to PpIX irradiance are not given, and a median PpIX-effective light dose of 18 J cm^−2^ was reported over all the treatments carried out in the three-year period. This retrospective view of dosimetry highlights the usefulness of such personal light meters and the authors importantly emphasised that appropriate calibration of the devices against daylight was necessary for accurate dosimetry.

Calibration and conversion from illuminance to PpIX-effective light dose can be complex. To circumvent the wavelength dependency issues with measuring illuminance, the Copenhagen wristwatch device was modified so that its spectral sensitivity matched the PpIX absorption spectrum [14]; this enables PpIX-weighted irradiance to be measured directly by the device. Details of the responsivity of this device are sparse, and similar caution should be exercised as was previously discussed in relation to the dosimetry method of Räsänen et al. [20]. The device has been used in two clinical studies and in one dose measurement study. In the first of these multi-centre studies, a mean PpIX-effective light dose of 9.4 J cm^−2^ (range 0.2–28.3) was measured for all patients (*n* = 119). All weather conditions, except rain, were permitted and there was no relationship between light dose and efficacy. The second of these studies measured mean PpIX-effective light doses of 6.5 ± 4.5 J cm^−2^ for patients in Norway and Sweden, and 9.6 ± 7.2 J cm^−2^ for those treated in Denmark. Considering all patients, a minimum PpIX-effective light dose threshold was found at 3.5 J cm^−2^.

#### 2.2.3. Clinical Relevance of Dosimetry in DPDT

**Figure 3:**
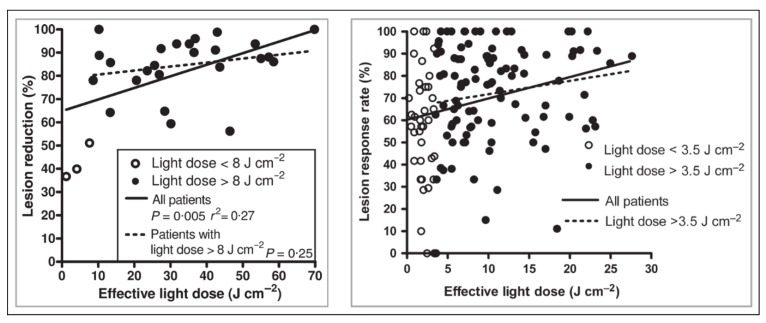
From [8,9]. Left, minimum PpIX-effective light dose threshold of 8 J cm^−2^ shown for patients with grade I AK lesions. Right, minimum PpIX-effective light dose threshold of 3.5 J cm^−2^ shown for all grades of lesion.

It has been suggested that a minimum threshold PpIX-weighted dose exists below which efficacy decreases and above which there is no further benefit to efficacy with increasing doses of light, thus that there is not dose-dependency once the threshold dose has been achieved. To date, only two studies have demonstrated a minimum threshold dose for effective DPDT: the studies by Wiegell et al. in 2009 and 2012 (Figure 3).

In the first of these studies, a relationship between dose and efficacy was found for all patients (*n* = 30), although when patients who received a PpIX-effective light dose < 8 J cm^−2^ were excluded, no significant association was seen between dose and efficacy (*n* = 27, grade I lesions only) [8]. The second study revealed a similar relationship (*n* = 137), although the minimum threshold PpIX-effective light dose was 3.5 J cm^−2^ (*n* = 100) [9]. Although this is less than half the previous study, it is worth considering that in the 2009 study, the mean treatment duration was 244 min, whereas in the 2012 study, it was 111 min.

It is noted that measurements on the wrist may represent approximately 50% of the dose received on the scalp [1,8,36,37], and this should be taken in to consideration when interpreting these data and when using such devices (e.g., 8 J cm^−2^ measured on the wrist = 16 J cm^−2^ on the head). This relationship was originally obtained for wrist watches measuring ultraviolet-B (UVB)—the same relationship is assumed for the longer and primarily visible wavelengths measured here, although the differing nature of direct and diffuse (light scattered from clouds and physical surroundings) proportions of UVB compared to even long wavelength ultraviolet-A (UVA) perhaps call for a similar study to be carried out using the PpIX wrist watches. In the study by Thieden et al. [36], the coefficient of variation (ratio of the standard deviation to the mean) in UV exposures measured at the wrist was reported to be as much as 84%, indicating that there is strong variability in doses measured this way. Any twisting of the arm such that the angle of the device to the sun is changed will result in inaccurate correlation between PpIX-effective light dose on the treatment and measurement planes, and if the detector has a poor angular response, this will exacerbate this issue. Although this can be partially controlled during a clinical trial, yet again, in practice, if patients have freedom of movement, then this becomes more important as an influencing factor.

This highlights the importance of the orientation of the measurement device during treatment. Dose relationships between different planes of orientation for specific devices are important to consider, particularly for personal dosimeters if mounted in a plane of orientation different to the treatment plane. Manley et al. illustrated the impact of having a treatment surface directed towards the sun at various times of the year [30]. They showed that during mid-winter, by laying a detector in the horizontal plane the PpIX-effective light dose delivered to the skin may be underestimated by more than four-fold if the treatment surface were directed towards the sun. Horizontal plane measurements are most common in DPDT, but this study shows the importance of the plane of orientation in dosimetry and patient treatment.

Subsequent studies have not shown any correlation between PpIX-effective light dose and efficacy, although studies have shown PpIX-effective light doses as low as 0.2 J cm^−2^ with successful treatment [14]. This absence of an apparent threshold PpIX-effective light dose in these later studies does not necessarily preclude the existence of a threshold PpIX-effective light dose, rather, it is perhaps a reflection of the many different ways that dose in DPDT has been measured, or that in these studies, the minimum PpIX-effective light dose threshold was not met.

In the 2009 study by Wiegell et al. where a minimum PpIX-effective light dose threshold was first observed, data on correlations between efficacy subdivided by lesion grading and PpIX-effective light dose were not shown [8]. In the 2012 study, this analysis was included yet showed no significant trends, although given the different levels of non-significance between grade I (*P* = 0.15), grade II (*P* = 0.12) and grade III (*P* = 0.78) AK, it is certainly worthwhile information to have, especially as grade III AK lesions were shown to have a significantly lower clearance rate after DPDT [9]. It is therefore important to understand what the minimum PpIX-effective light dose means in the context of lesion grade. In these two studies, the classification for grading AK lesions defined by Olsen et al. was applied [38], though grading for AKs may be carried out using other systems such as that proposed by Röwert-Huber et al. or the AKASI [39], [40]. Unsurprisingly, there is no clear consensus on the relationship between lesion grade and the minimum PpIX-effective light dose threshold required for effective disease clearance.

Although previous studies had shown the possibility of successful treatment with lower PpIX-effective light doses, the European consensus still opted to conservatively choose 8 J cm^−2^ as the recommended minimum PpIX-effective light dose (un-weighted solar irradiance of 130 W m^−2^ with 2 h exposure) [2]. Importantly, the authors state that the dose of light should be delivered over a duration of at least 2 h, although treatment times as low as 1.5 h have been shown, with no observable reduction in efficacy [14]. Delivering the same dose over a much shorter duration (e.g., 30 min) does not necessarily equate to as efficacious a treatment. There are other mechanisms involved in DPDT beyond light irradiation, such as photosensitiser production and oxygen depletion, so reciprocity should not be assumed.

### 2.3. Dose Measurements by Location

Some studies have carried out dosimetry at various locations in order to ascertain where and when DPDT might be suitable. The first such study measured PpIX-effective light dose across six international locations, from Iceland to Israel [22]. PpIX dosimeter watches were sealed in weatherproof containers and recorded data at each of the locations during the latter half of 2008. As an extra treatment criterion, an ambient temperature of no less than 10 °C is recommended based on the clinical experience of the authors. Using these criteria, which include a minimum PpIX-effective light dose threshold of 8 J cm^−2^, treatment was deemed possible on nearly all days from July until September to October, depending on the latitude of the location. The authors in this study noted that neither UV Index nor temperature or weather conditions were suitable for predicting PpIX-effective light dose.

In a follow-up publication to the Australian study [3], irradiance values were determined through radiative transfer models (Meteonorm; Meteotest, Bern, Switzerland) for eight locations in Australia in order to determine the suitability of DPDT in these locations throughout the year. Meteonorm is a combination of ground-based measurements, interpolation and predictive modelling [41,42].

The authors declared a minimum induced clinical benefit (MICB) of 40 W m^−2^, with no PpIX weighting, as there was no correlation observed between treatment efficacy and mean irradiance from the previous [15] Australian study (mean 305.8 W m^−2^, range 40–585 W m^−2^). This does not indicate that below 40 W m^−2^ DPDT would not be effective, only that measurements below this MICB were not made, so no direct inferences can be made with regards to a minimum effective dose. They found that in all locations in Australia, treatment is possible year-round, assuming a 2-h treatment duration and tolerable outdoor conditions. This has proven to be a somewhat useful method of determining irradiance as the practitioner is not required to take measurements directly.

Indeed, Meteonorm has been used in other studies. Grinblat et al. used the same method to determine irradiance across multiple locations in South America and Mexico in order to assess year-round suitability of DPDT and demonstrated that all the measurements were above the MICB of 40 W m^−2^ [23]. In the 2015 European consensus [2], Morton et al. reported on irradiances across several European locations and compared the to critical values (min, max, mean) obtained in the studies by Lacour (2015) and Wiegell (2009) [4,8]. They demonstrated that DPDT was feasible year-round in Southern Europe (37–43° N), and progressively less so towards more northern locations (60° N, March to September), although the authors pointed out that this only accounts for PpIX-effective light dose and not the tolerability of outdoor weather conditions.

O’Mahoney et al. considered the suitability of DPDT across several locations in the UK and Ireland [24] using an illuminance-to-PpIX irradiance conversion model derived from spectral irradiance measurements (Glacier X, BWTek, Newark, NJ, USA) taken in Chilton, UK (51.575° N, 1.318° W). This model was verified in other UK locations and then applied to historic illuminance measurements in order to determine PpIX-effective light dose in each location. The authors compared the data against a minimum threshold PpIX-effective light dose of 4 J cm^−2^, derived conservatively from a variety of studies [8,9,17,18], and reported that DPDT would be suitable in many locations in the UK and Ireland throughout much of the year and was limited primarily by temperature and precipitation, as has been suggested previously [22]. Conservatory-based DPDT was also considered, having first been suggested by Lerche et al. in Copenhagen [43]. The range of months of the year in which conservatory-based DPDT is possible was greater than DPDT alone. A subsequent study by Manley et al. independently verified this method as appropriate for DPDT dosimetry [30].

Recently, McLellan et al. documented UV exposure levels experienced during suitable times for DPDT [44]. Data from Public Health England’s monitoring network provided erythema-weighted UV (UVE), UVA irradiance and illuminance data [45], from which suitable times of the year in 12 UK and international locations were determined and the corresponding UV doses were outlined. This work showed that therapeutically relevant PpIX-effective light doses (assuming > 4 J cm^−2^ is needed) are possible when UV exposures are minimal. It may have particular relevance for high-risk patients (e.g., immunosuppressed patients) with field-change AK, for whom it is most important to keep UV exposure levels to a minimum. Ultimately, this is important published evidence which adds to the wealth of knowledge relating to DPDT and helps to strengthen the confidence of clinicians, practitioners and patients with respect to the UV and visible light exposure during DPDT.

### 2.4. Sunscreens and PpIX-Effective Light Dose

Wiegell et al. showed that there was very little overlap between the absorption profiles of PpIX and an SPF20 organic sunscreen (P20^®^, Riemann A/S, Hilleroed, Denmark) [8], and in 2015, work by Galderma investigated photobleaching of PpIX in ex vivo skin samples with and without sunscreen [46]. After one hour of irradiation by a solar simulator, the ex vivo skin samples were analysed for remaining concentration of PpIX by high performance liquid chromatography (HPLC), and the authors demonstrated that there was no significant difference in PpIX photobleaching between samples with or without sunscreen applied, whilst there was significant photobleaching compared to a non-irradiated sample.

However, it was recently demonstrated that sunscreens may impact on PpIX-effective light dose more than had previously been considered [47]. This study was conducted by measuring the transmission of several sunscreens using a sunscreen analyser (Labsphere UV-1000, Labsphere, North Sutton, NH, USA) and applying the transmission spectra to PpIX-weighted daylight in order to obtain the percentage reduction in dose as a result of light attenuation by sunscreens. In contrast to the previous work, it was shown that there was a significant reduction in PpIX-effective light dose with each sunscreen, indicating that sunscreen application may reduce effective dose delivery in DPDT and emphasising that sunscreen choice had the potential to impact DPDT efficacy. It is suggested that the changes in sunscreen formulations over the years may explain, at least in part, the variability of the outcomes and conclusions of these studies, but the potential influencing factor of sunscreen choice should be kept in mind and it would be prudent to consider further how sunscreens affect light transmission in future studies on PpIX-effective light dose.

## 3. Conclusions

Whilst the majority of DPDT users are keen to undertake DPDT without real-time measurements of light exposure, this can only be done with confidence if based on a robust understanding of the methods and techniques involved in accurate DPDT dosimetry, the guidance and recommendations that arise from such studies. There are several different methods of measuring PpIX-effective light dose for DPDT, each with their own advantages and disadvantages. In terms of practicality, simplicity and convenience, the PpIX-effective light dose wristwatch developed in Copenhagen is perhaps the optimum choice, assuming appropriate calibrations to daylight and body site corrections are performed. While there are scant technical details in the published literature, the device is stated to be calibrated to spectral irradiance measurements of daylight and has a good cosine response. Moreover, the device provides direct measurements of PpIX-effective light dose and thus, there is less of a burden on the user for processing data. Additionally, personal devices such as these wristwatches or other personal light meters [35] do, perhaps, more accurately represent actual patient exposure, rather than a single centralised measurement system representing exposure for many different patients, if combined with a correction based on body site. This is particularly relevant if patients have freedom of movement during treatment rather than being confined to hospital gardens for example.

Dosimetry in DPDT is understood to be important clinically, however the specific parameters required for effective clearance of AKs are not extensively characterised. Several studies have sought to clarify the requirements regarding PpIX-effective light dose, although variation in techniques and patient populations make the task much more complex than initially apparent. What is clear, however, is that there is very little published information on the link between PpIX-effective light dose and treatment efficacy, highlighting the need for robust light dosimetry in such studies. It is therefore important that future studies and practices carry out dosimetry where possible and make these measurements open, accessible and detailed in order to maximise the effective delivery of DPDT for clinicians and patients and to fully elucidate the link between PpIX-effective light dose and treatment efficacy.

## Figures and Tables

**Table 1:** Summary of DPDT studies carrying out dosimetry, and studies investigation location-based dosimetry analysis.

Author (et al.)	Year	Study Type	Dosimetry Method	PpIX Spectrum Source	Sunscreen	Time (hours)	Light Dose (J cm^−2^)	Range
**Wiegell** [12]	2008	RCT	Luxmeter informs radiative transfer model	[13]		2.5	43.2	11.7–65.9
**Wiegell** [8]	2009	RCT	Wristwatch luxmeter informs radiative transfer model	[13]	P20	4	30.1	1.2–69.8
**Wiegell** [14]	2011	RCT	Wristwatch (PpIX-weighted)	[13]	P20	2 or 3	9.4	0.2–28.3
**Wiegell** [9]	2012	RCT	Wristwatch (PpIX-weighted)	[13]	P20	1.5 or 2.5	6.5 and 9.6	
**Rubel** [15]	2014	RCT	Spectroradiometer	N/A	‘High SPF’	2	22.8	3–46
**Grinblat** [16]	2014	Non-RCT	Pyranometer (Irradiance)	N/A	‘SPF30’	1–1.5	28.25	7.9–45.3
**Lacour** [4]	2015	RCT	Spectroradiometer	N/A	Actinica	2	267 (W m^−2^)	44–601 (W m^−2^)
**Togsverd-Bo** [17]	2015	RCT	Illuminance	N/A	P20	2	48773 (lx)	10600–86357 (lx)
**O’Gorman** [18]	2016	RCT	Irradiance, illuminance and spectral irradiance.	N/A	P20	2	21.38	3.2–43
**Nissen** [19]	2017	RCT	Illuminance	N/A	P20	2	36477 (lx)	1322–94234 (lx)
**Räsänen** [20]	2018	RCT	Irradiance	N/A	P20	2	12.89	5.96–17.93
**Heerfordt** [21]	2019	RCT	Illuminance	N/A	N/A	2	38640 (lx)	7200–85200 (lx)
**Wiegell** [22]	2013	Dose measurement	Wristwatch (PpIX-weighted)	[13]		2		
**Morton** [2]	2015	Dose measurement	Meteonorm (Radiative transfer outputs irradiance)	N/A				
**Spelman** [3]	2015	Dose measurement	Meteonorm (Radiative transfer outputs irradiance)	N/A				
**Grinblat** [23]	2016	Dose measurement	Meteonorm (Radiative transfer outputs irradiance)	N/A				
**O’Mahoney** [24]	2017	Dose measurement	Spectroradiometer and illuminance	[25]		2		
